# Association between exposure to ambient air pollution, meteorological factors and atopic dermatitis consultations in Singapore—a stratified nationwide time-series analysis

**DOI:** 10.1038/s41598-024-60712-4

**Published:** 2024-05-06

**Authors:** Diyar Mailepessov, Janet Ong, Muhammad Zafir Mohamad Nasir, Joel Aik, Maye Woo, Xiahong Zhao, Hong Liang Tey, Yik Weng Yew

**Affiliations:** 1 Environmental Health Institute, National Environment Agency, 11 Biopolis Way #06-05/08, Helios Block, Singapore, 138667 Singapore; 2 Pre-Hospital and Emergency Research Centre, Duke-NUS Medical School, 8 College Road, Singapore, 169857 Singapore; 3 Environmental Quality Monitoring Department, Environmental Monitoring and Modelling Division, National Environment Agency, Singapore, 228231 Singapore; 4 National Skin Centre, Singapore, Singapore; 5 Lee Kong Chian School of Medicine, Nanyang Technological University, Singapore, Singapore; 6 Yong Loo Lin School of Medicine, National University of Singapore, Singapore, Singapore

**Keywords:** Climate sciences, Environmental sciences, Health care

## Abstract

Atopic dermatitis (AD) is a chronic inflammatory skin disease affecting approximately 20% of children globally. While studies have been conducted elsewhere, air pollution and weather variability is not well studied in the tropics. This time-series study examines the association between air pollution and meteorological factors with the incidence of outpatient visits for AD obtained from the National Skin Centre (NSC) in Singapore. The total number of 1,440,844 consultation visits from the NSC from 2009 to 2019 was analysed. Using the distributed lag non-linear model and assuming a negative binomial distribution, the short-term temporal association between outpatient visits for AD and air quality and meteorological variability on a weekly time-scale were examined, while adjusting for long-term trends, seasonality and autocorrelation. The analysis was also stratified by gender and age to assess potential effect modification. The risk of AD consultation visits was 14% lower (RR_10th percentile_: 0.86, 95% CI 0.78–0.96) at the 10th percentile (11.9 µg/m^3^) of PM_2.5_ and 10% higher (RR_90th percentile_: 1.10, 95% CI 1.01–1.19) at the 90th percentile (24.4 µg/m^3^) compared to the median value (16.1 µg/m^3^). Similar results were observed for PM_10_ with lower risk at the 10th percentile and higher risk at the 90th percentile (RR_10th percentile_: 0.86, 95% CI 0.78–0.95, RR_90th percentile_: 1.10, 95% CI 1.01–1.19). For rainfall for values above the median, the risk of consultation visits was higher up to 7.4 mm in the PM_2.5_ model (RR_74th percentile_: 1.07, 95% CI 1.00–1.14) and up to 9 mm in the PM_10_ model (RR_80th percentile_: 1.12, 95% CI 1.00–1.25). This study found a close association between outpatient visits for AD with ambient particulate matter concentrations and rainfall. Seasonal variations in particulate matter and rainfall may be used to alert healthcare providers on the anticipated rise in AD cases and to time preventive measures to reduce the associated health burden.

## Introduction

Atopic dermatitis (AD), commonly known as eczema, is a chronic inflammatory skin condition often associated with redness of the skin and formation of small blisters which are often accompanied by itchy sensations^1,2^. The International Study of Asthma and Allergies in Childhood (ISAAC) reported that ADHD can affect as much as 20% of children across the globe^3^. AD patients incur both direct (hospitalization bills, medications)^4,5^ and indirect costs (loss of work hours, transportation cost to specialist clinics)^6^. The patient’s quality of life such as mental health^7,8^, sleep pattern^9^, and lifestyle can also be adversely affected. As such, there is significant interest in better understanding and identifying the associated risk factors.

Known risk factors for the occurrence of AD include deficiencies in immune mechanisms (decrease of antimicrobial peptides) and genetics (loss of filaggrin, an important epidermal protein)^2,10^. Environmental exposure to air pollutants, specifically particulate matter was found to be associated with AD in several original publications and reviews^11–13^. Additionally, a recent study suggested that environmental factors such as temperature, humidity, and rainfall modified the relationship between particulate matter exposure and the AD^14^. In Asia, studies conducted in China, South Korea and Taiwan showed that increase in particulate matter, along with nitrogen dioxide (NO_2_) and sulfur dioxide (SO_2_) was associated with increased consultation visits for AD^15–17^. However, studies investigating relationship between particulate matter and other air pollutants and AD in tropical countries in Asia are rare compared to temperate settings^18^.

Furthermore, different demographic groups within the society may display differing tendencies in developing AD^19,20^. Exposure to air pollutants is reported to affect men and women differently where hormones could play varying roles in the transport and accumulation of toxic substances around the body^21^. Most studies target young children with the assumption that AD is a paediatric disease and does not affect adults^22^. However, recent studies have reported an increased prevalence among adults who develop AD during adulthood^15,23^. A study of children in Taiwan showed that females were more susceptible to AD in association with increased air pollutant concentrations compared to males^24^. There are also associations between air quality and the prevalence of AD, where an increase in concentrations of airborne pollutants (PMs and NO_2_) saw a corresponding increase in AD symptoms among young children in Seoul^16,17^.

In a cohort of Singapore patients, 61.2% of participants experienced the onset of AD during their childhood before the age of 10^25^. A comparative study of two ISAAC surveys conducted seven years apart (1994 and 2001) in Singapore showed an increased prevalence of AD among children (6–7 and 12–15 years old)^26^. The quality of life of young children in Singapore with AD was found to be greatly affected, to different degree based on gender and race^27,28^. However, there are to our knowledge no published studies on associations between air quality, weather and the incidence of AD outpatient visits conducted in Singapore.

We sought to understand the association between air quality and meteorological variations with AD consultation reports in Singapore to inform healthcare resource allocation plans and the timing of advisories aimed at reducing the health burden during anticipated peak periods. The time-series study includes both children and adults to provide a robust overview of the onset of AD in association with air quality variations in Singapore. We hypothesised that increases in ambient air pollutants adjusted for meteorological variables, were positively associated with the number of AD reports.

## Methods

### Ethics statement

This study was approved by the Environmental Health Institute of the National Environment Agency (NEA), Singapore (TS277).

### Study setting

Located in Southeast Asia, Singapore is a city-state with an estimated population of 5.4 million people^29^. Singapore experiences a warm tropical climate characterized by high rainfall and humidity throughout the year^30^. The National Skin Centre (NSC) is the largest outpatient dermatological centre in Singapore. It is situated in the middle of the island nation and consistently manages more than 50% of all dermatological referrals in the country. NSC has an annual patient attendance of 175,173 in 2020^31^ and the most common condition managed in NSC over the study period was eczema.

### Outcome measure

We obtained reports of all AD outpatient consultations (n = 1,440,844) from NSC from 2009 to 2019. These reports represented patients who were diagnosed with AD by dermatologists based on ICD9 and SNOWMED diagnosis codes. We included all new episodes of AD and the follow up visits were excluded from the analysis. The reports included the date of onset of illness, as well as demographic information, such as gender and age. We classified the patients into four age groups: < 7 years old, 7–12 years old, 13–18 years old, and > 18 years old, which corresponded to toddler/preschool, childhood, adolescent and adult respectively, similar to the study conducted by Ng et al.^18^ with the exception of an additional youngest group. We aggregated the AD reports by epidemiological week to analyse the outcome measure on a weekly timescale over the entire study duration.

### Ambient air quality and climate exposure data

The NEA monitors ambient air quality continuously with 22 remote sensors deployed across the island. We obtained from the NEA daily measures of ambient concentrations of particulate matter (PM) of aerodynamic diameter less than 2.5 μm (PM_2.5_) and aerodynamic diameter less than 10 μm (PM_10_), ozone (O_3_), sulphur dioxide (SO_2_), nitrogen dioxide (NO_2_) and carbon monoxide (CO) over the study duration and obtained their daily measures averaged over each week.

The NEA has a network of meteorological observing stations that provide real-time observations across Singapore. These stations have rainfall, temperature, and humidity sensors which are collated centrally for reference. We obtained local climate data from 11 mainland weather stations located across the island and used the arithmetic mean of climate data across all stations to derive weekly mean measures of ambient temperature, and rainfall. The weekly mean measures of absolute humidity were derived from measures of mean temperature and relative humidity. As relative humidity was observed to high correlation with mean temperature (r = − 0.72), absolute humidity was used in the study (r = 0.24).

### Statistical analyses

We analysed the short-term association (i.e., immediate effects at lag week 0 and delayed effects from lag week 1–6) between consultation visits for AD with air quality and meteorological exposures on a weekly timescale. Longer lag effect of 6 weeks was included to account for a relatively long-lasting effect of air pollutants exposure on AD onset and exacerbation as suggested in a previous study^32^. A previous study has reported a non-linear relationship between AD and air pollutants and meteorological variables^33^. We therefore used the distributed lag non-linear model (DLNM) framework, which accounts for both non-linear and delayed associations between the exposure and response variables simultaneously, to investigate the temporal association of our exposure variables on weekly number of consultation visits over the maximum lag period of 6 weeks^34^. We accounted for over-dispersion in the outcome measure by using a negative binomial regression model with a population offset term. We adjusted for long-term trends and seasonal fluctuations in AD reports using natural cubic splines with 7 degrees of freedom (*df*) per year^35^. Public holidays may be associated with differential health-seeking behaviour that may affect consultation visits. To account for that, we included public holidays a variable denoting number of public holidays in the week. We first developed a core model that examines the effect of all meteorological variations on a number of consultation visits and subsequently added air pollutant variables to assess their effects.

The inclusion of highly correlated independent variables may lead to inaccurate effect estimates. We used Pearson correlation coefficients to assess potential collinearity between air pollutants, and a correlation coefficient of 0.5 was used as the maximum threshold between two air pollutants (Supplementary material, Table S1)^36^. To avoid multi-collinearity, we have built two separate core models for PM_2.5_ and PM_10_ as these two variables were highly correlated (correlation coefficient = 0.98). The core models included either PM_2.5_ or PM_10_, and a term for public holidays, adjusted for long term trend using cubic splines described above and an offset term to account for population size changes (Supplementary material, Table S2). We then tested the addition of each air pollution and meteorological variable to these core models using log-likelihood ratio (LRT) test and only retained variables with statistically significant LRT test outcome (Supplementary material, Table S2). We examined the Partial Autocorrelation Function (PACF) (Supplementary material, Figs. S1 and S2) plots of the penultimate model and added lags of deviance residuals corresponding to the degree of residual autocorrelation observed to account for serial autocorrelation.

The final PM_2.5_ model, as well as PM_10_ models which were fitted separately as they were correlated, are shown in Eqs. (1) and (2):

1 $$\begin{aligned} \log E\left( {Y_{t} } \right) & = \beta_{0} + ns\left( {t,df} \right) + \beta_{1} holidays + S\left( {AH_{t} , \upsilon , \tau } \right) + S\left( {Rf_{t} ,\omega ,\tau } \right) + S\left( {\left( {PM_{2.5} } \right)_{t} ,\gamma ,\tau } \right) \\ & \quad + \beta_{1} \mathop \sum \limits_{l = 1}^{l = L} res_{l} + \log \left( {N_{t} } \right) \\ \end{aligned}$$

2 $$\begin{aligned} \log E\left( {Y_{t} } \right) & = \beta_{0} + ns\left( {t,df} \right) + \beta_{1} holidays + S\left( {AH_{t} ,\upsilon ,\tau } \right) + S\left( {Rf_{t} ,\omega ,\tau } \right) + S\left( {\left( {PM_{10} } \right)_{t} ,\gamma ,\tau } \right) \\ & \quad + \beta_{1} \mathop \sum \limits_{l = 1}^{l = L} res_{l} + \log \left( {N_{t} } \right) \\ \end{aligned}$$

where log *E(Y*_*t*_*)* is the expected number of weekly consultation visits on a week t with a log form and *β*_*0*_ is the intercept. The seasonality and long-term trend are adjusted using natural cubic splines *ns(t, df)*. The cross basis forms of all air quality and meteorological variables are represented in the form of *S(Vart, x, τ)* where *Var*_*t*_ denotes the variable on the week t, *x* is the coefficient of a vector of that variable, and *τ* the number of lagged weeks for the relationship between the outcome and the variable. To adjust for the population size effect at different times, an offset term log(*Nt*) representing logged weekly population estimates interpolated from the mid-year annual population census data was added. The residual autocorrelation is represented by the sum of deviance residual—*resl* with coefficient *β*_*1*_.

Using PM_2.5_, we also assessed for potential gender and age effect modification. Since PM_2.5_ is a subset of PM_10_ and both are highly collinear (r = 0.98), the gender and age effect modification are likely to be similar reducing the need for examining these relationships in both models.

We reported the cumulative relative risk (RR) of reported AD consultation visits at the 10th and the 90th and other important percentiles for each climatic and air quality variable compared to its median with corresponding 95% confidence intervals (95% CI). All statistical tests were two-tailed, and *p* values less than 0.05 were considered statistically significant. All statistical analyses were performed using R version 3.6.3.

## Results

### Descriptive statistics

**Figure 1:**
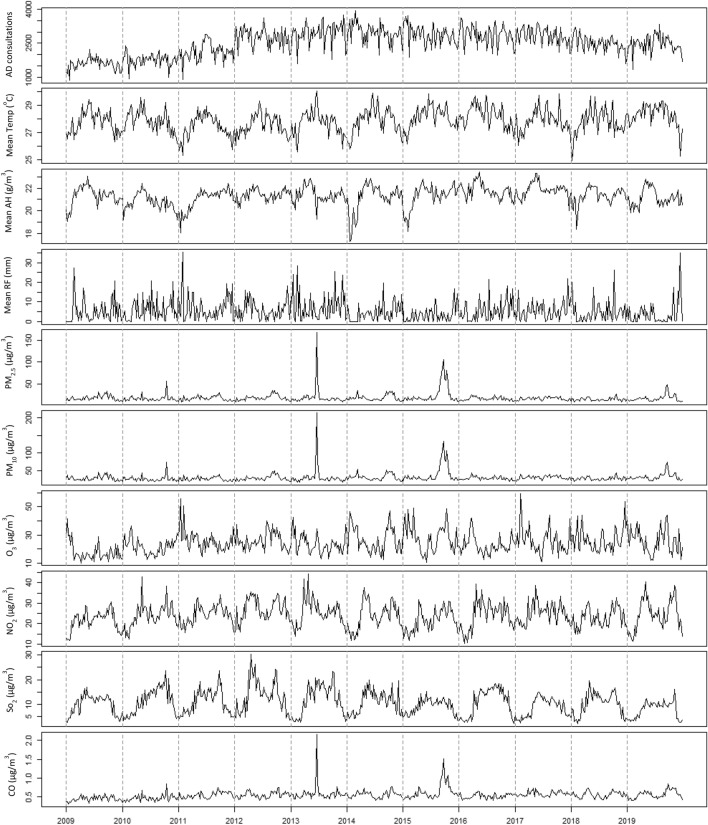
Weekly measures of AD consultation visits, meteorological factors and air pollutants from 2009 to 2019. Temp denotes temperature, RF denotes rainfall, AH denotes absolute humidity.

There was a total of 1,440,844 reported AD consultations to NSC over the study period from 2009 to 2019. The weekly number of AD consultations ranged between 861 and 3944, with an average of 2,519 (Table 1). Among the four age groups considered, patients aged 13–18 years constituted the highest number (Table 2), however the difference between age groups was not statistically significant. There was no significant difference in AD incidence between sexes. There was no obvious seasonal variation in AD consultation visits over the years, while some seasonal variations were in the weekly mean temperature and absolute humidity throughout the year (Fig. 1). Likewise, air pollutants displayed seasonal variations with occasional spikes in the concentrations (Fig. 1). PM_2.5_, PM_10_ and CO concentrations were highly correlated over the study period (r > 0.7, *p* < 0.05), while NO_2_ was highly correlated with SO_2_ (r > 0.6, *p* < 0.05) (Table S1).

**Table 1:** Summary of consultation visits, meteorological and air quality factors in Singapore, 2009 to 2019.

Variable	Weekly measures					
	Mean	SD	Median	IQR	Min	Max
Consultations for atopic dermatitis	2519	603.0	2608	2038 – 2992	861	3944
Meteorological factors						
Mean temperature (°C)	27.9	0.9	28.0	27.3–28.4	25.0	30.1
Mean absolute humidity (g/m^3^)	21.3	0.9	21.4	20.9–21.9	17.3	23.5
Mean rainfall (mm)	5.3	5.6	3.7	0.0–7.8	0.0	35.5
Air quality						
PM_2.5_ (µg/m^3^)	18.4	10.8	16.1	13.5–19.7	9.2	167.0
PM_10_ (µg/m^3^)	30.2	13.2	28.0	24.2–32.4	16.7	214.0
SO_2_ (µg/m^3^)	10.5	5.0	10.6	6.0–13.6	2.2	30.2
NO_2_ (µg/m^3^)	23.9	5.9	23.8	20.0–28.0	10.3	44.3
O_3_ (µg/m^3^)	24.4	8.0	23.1	18.3–29.1	10.0	59.6
CO (mg/m^3^)	0.5	0.1	0.5	0.5–0.6	0.3	2.2

SD refers to standard deviation, IQR refers to interquartile range, while min and max to minimum and maximum, respectively.

**Table 2:** Incidence of AD by sex, age group and year.

	Annual AD incidence per 100 population (95% CI)
Overall	24.1 (16.0, 32.2)
Sex	
Female	32.8 (21.4, 44.3)
Male	34.6 (21.3, 48.0)
Age group	
< 7 years old	23.3 (1.5, 45.1)
7–12 years old	25.8 (7.0, 44.7)
13–18 years old	33.3 (15.4, 51.3)
> 18 years old	26.6 (19.1, 34.2)
Year	
2009	2.04 (2.03, 2.05)
2010	2.29 (2.28, 2.30)
2011	2.80 (2.78, 2.82)
2012	3.66 (3.64, 3.68)
2013	3.93 (3.91, 3.96)
2014	4.06 (4.04, 4.08)
2015	3.89 (3.87, 3.91)
2016	3.92 (3.90, 3.94)
2017	3.88 (3.86, 3.89)
2018	3.44 (3.42, 3.45)
2019	3.43 (3.41, 3.45)

### Meteorological and air quality effects

**Figure 2:**
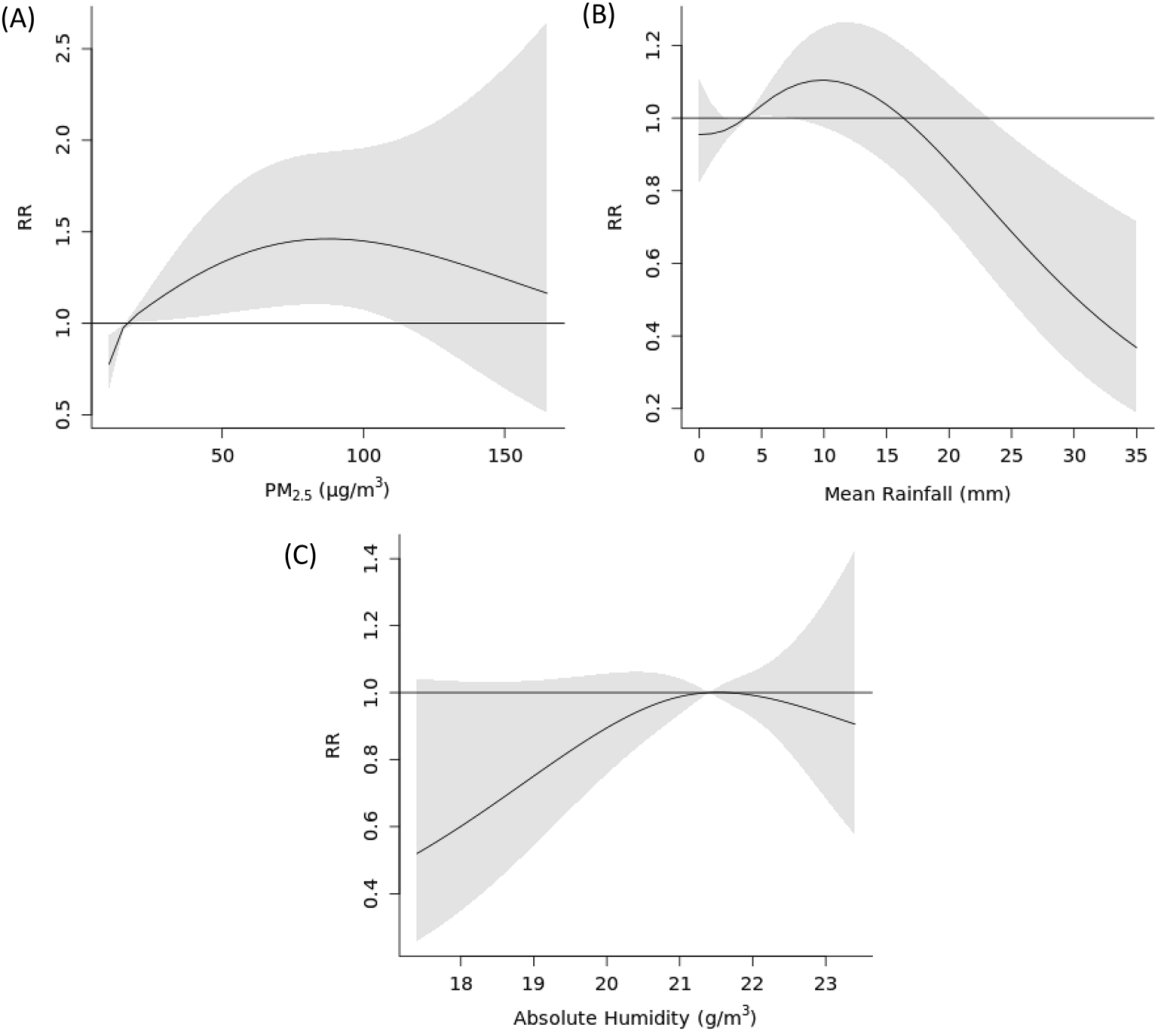
Exposure–response curve showing the overall cumulative effect of (**A**) PM_2.5_ (µg/m^3^), (**B**) Mean Rainfall (mm), and (**C**) Absolute Humidity (g/m^3^) on consultation visits, with reference to their median values observed in PM_2.5_ model. Solid lines represent relative risk (RR), and grey shaded areas represent 95% confidence intervals (CI).

**Figure 3:**
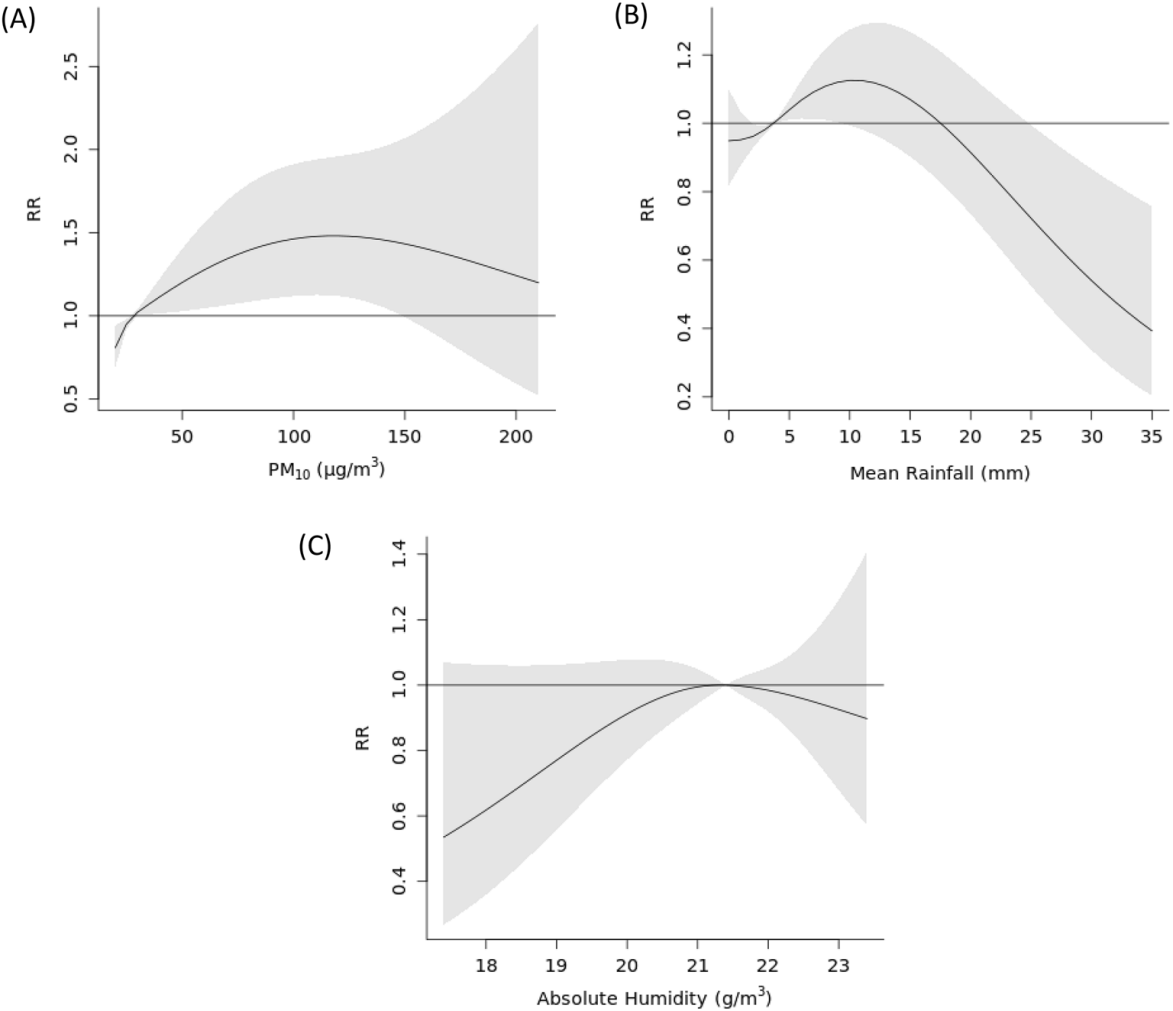
Exposure–response curve showing the overall cumulative effect of (**A**) PM_10_ (µg/m^3^), (**B**) Mean Rainfall (mm), and (**C**) Absolute Humidity (g/m^3^) on consultation visits, with reference to their median values observed in PM_10_ model. Solid lines represent relative risk (RR), and the grey shaded areas represent 95% confidence intervals (CI).

Among air pollutants only PM_2.5_ and PM_10_ were significantly associated with the number of AD consultation visits. This effect was consistent after accounting for long-term trend and seasonal fluctuations in the number of consultation visits and potential confounding effects of rainfall and absolute humidity in 2 separate models (Figs. 2, 3). For both PM_2.5_ and PM_10,_ statistically significant associations were found to be non-linear. Both air pollutants exhibited a similar inverted U-shape relationship with the number of consultation visits (Figs. 2A, 3A). The risk of AD consultation visits was 14% lower (RR_10th percentile_: 0.86, 95% CI 0.78–0.96) at the 10th percentile (11.9 µg/m^3^) of PM_2.5_ and 10% higher (RR_90th percentile_: 1.10, 95% CI 1.01–1.19) at the 90th percentile (24.4 µg/m^3^) compared to the median value (16.1 µg/m^3^). Similar results were observed for PM_10_ with lower risk at the 10th percentile and higher risk at the 90th percentile (RR_10th percentile_: 0.86, 95% CI 0.78–0.95, RR_90th percentile_: 1.10, 95% CI 1.01–1.19). Lag specific associations for both PM_2.5_ and PM_10_ and AD consultation visits can be found in Supplementary material (Supplementary material, Figs. S3, S4).

We found statistically significant non-linear association between rain and the risk of AD consultation visits. The association exhibited a reverse-S-shaped relationship, where the risk was higher for values immediately above the median value and lower for values from around the 25 mm mark in both PM_2.5_ and PM_10_ models (Figs. 2B, 3B). For PM_2.5_ and PM_10_, for values below the median, no statistically significant difference compared to the median was observed (RR_10th percentile_: 0.95, 95% CI 0.83–1.10, RR_10th percentile_: 0.95, 95% CI 0.83–1.09 for PM_2.5_ and PM_10_ models, respectively). For values above the median, the risk of consultation visits was higher up to 7.4 mm in the PM_2.5_ model (RR_74th percentile_: 1.07, 95% CI 1.00–1.14) and up to 9 mm in the PM_10_ model (RR_80th percentile_: 1.12, 95% CI 1.00–1.25). For extreme values above 25 mm, the risk of consultation visits was statistically significantly lower compared to the median value in both models. Lag specific associations for the relationship between rain and AD consultation visits can be found in Supplementary material (Supplementary material, Figs. S5, S6).

Association between absolute humidity and AD consultation visits was not found to be significant in cumulative plots (Figs. 2C, 3C), however, the individual lags at weeks 4 and 5 in both PM_2.5_ and PM_10_ models were observed to be significant (Supplementary material, Figs. S7, S8). At those lags the risk was lower for values below and above the absolute humidity median value.

No statistically significant associations were found between NO_2_, SO_2_, O_3_, CO, temperature variables and AD consultation visits (*p* < 0.05).

### Gender and age effect modification

**Figure 4:**
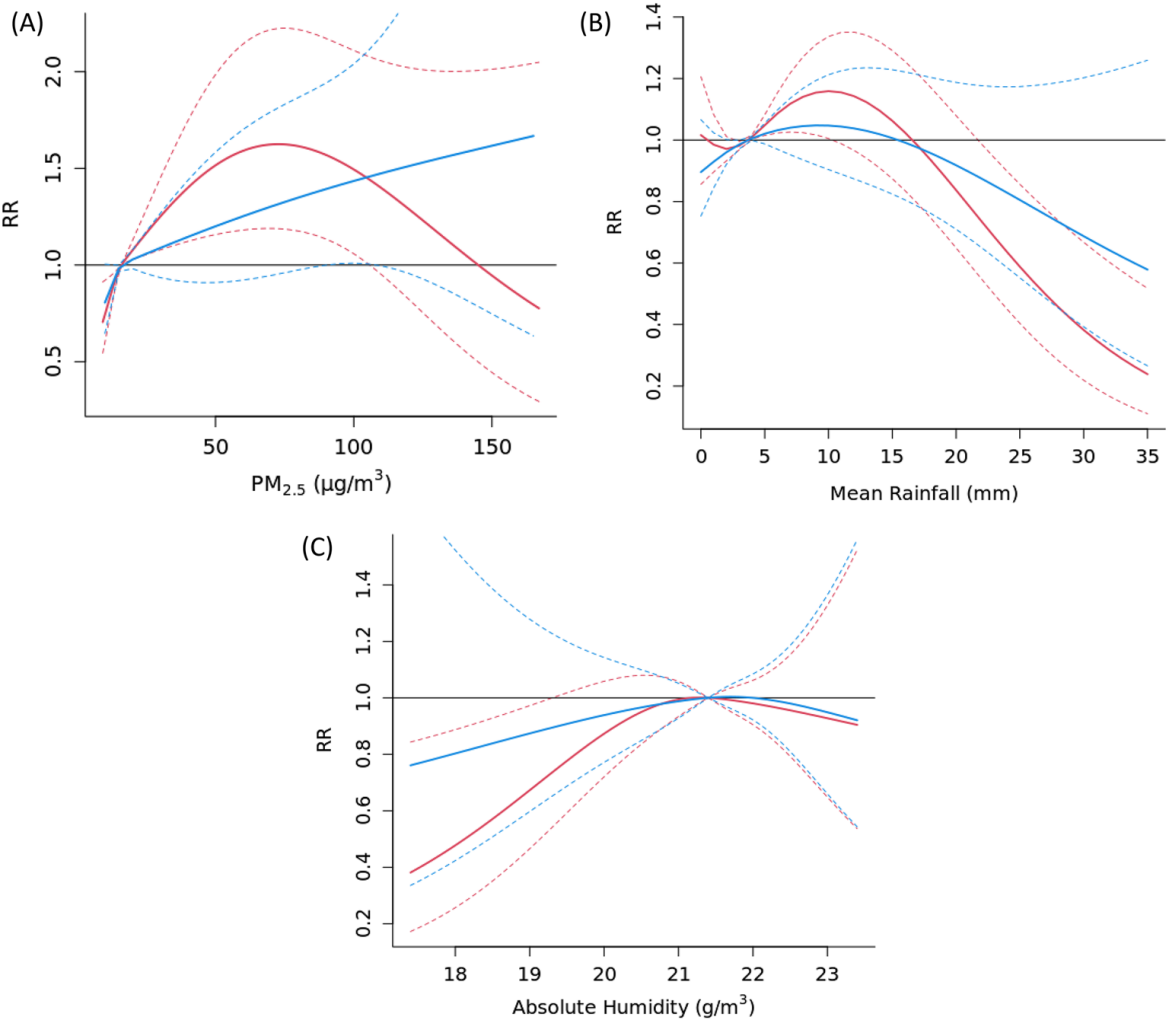
Exposure–response curve showing the overall cumulative effect of PM_2.5_ concentration (**A**), mean rainfall (**B**), and absolute humidity (**C**) on consultation visits in females (blue lines) and males (red lines), with reference to their median value. Solid lines represent relative risk (RR), and dashed lines represent 95% confidence intervals (CI).

**Figure 5:**
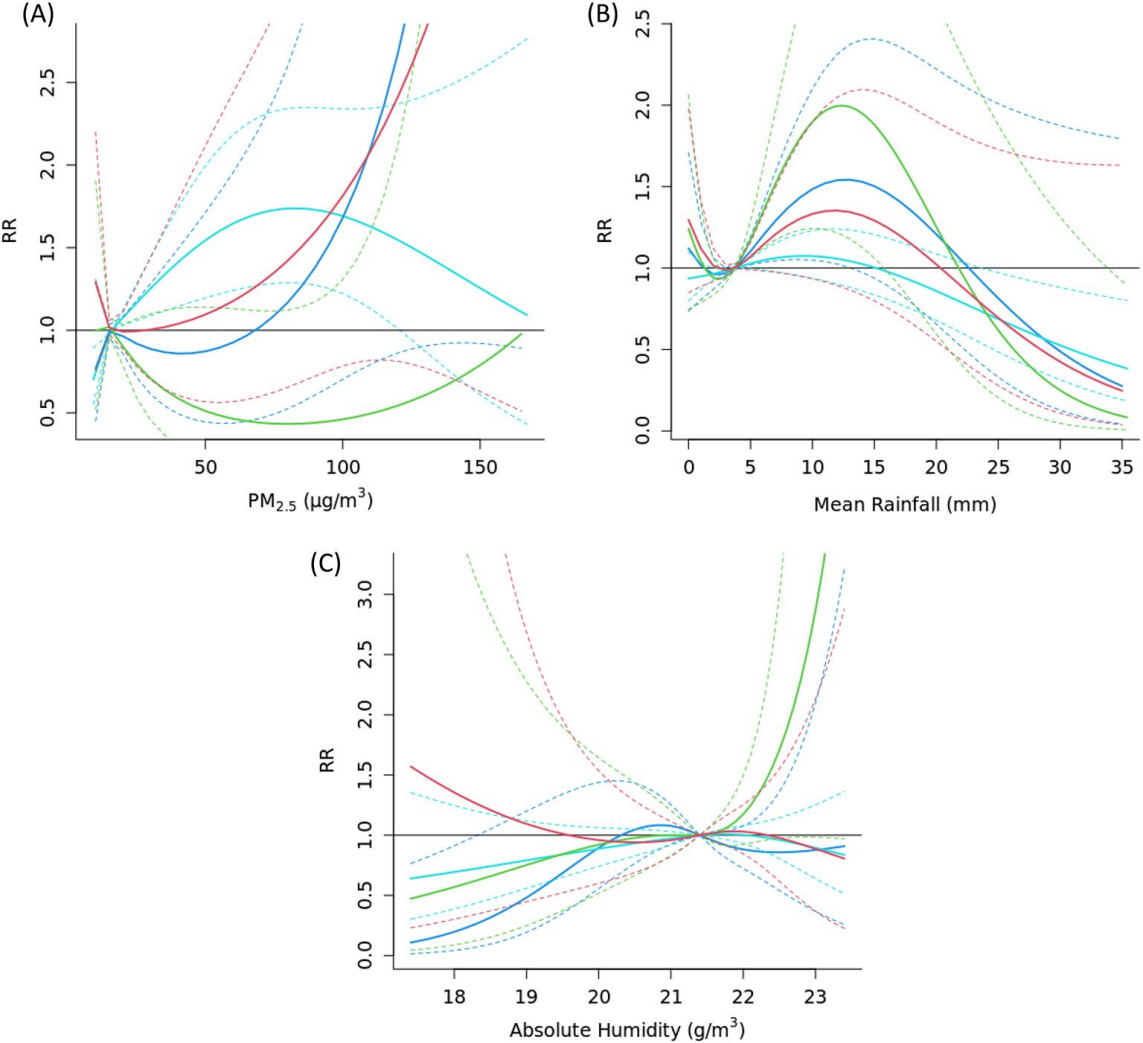
Exposure–response curve showing the overall cumulative effect of PM_2.5_ concentration (**A**), mean rainfall (**B**), and absolute humidity (**C**) on consultation visits in individuals aged less than 7 years (red), 8–12 years (green), 13–18 years (blue), and greater than 18 years old (cyan), with reference to their median value*s*. Solid lines represent relative risk (RR), and dashed lines represent 95% confidence intervals (CI).

We performed stratified analysis to assess potential sex and age effect modification using the PM_2.5_ model. We did not observe any statistically significant sex differences when we compared the dose–response relationships for PM_2.5_, AH and rainfall with AD consultation visits for male and female subgroups (Fig. 4). For the age modification, statistically significant difference was only detected for PM_2.5_ between age groups 18 and above and 7- to 12-year-olds (Fig. 5). No statistically significant interactions between age and PM_2.5_ were detected for rainfall and absolute humidity (*p* < 0.05).

## Discussion

In this study we sought to determine the effects of air quality and meteorological factors on the number of AD consultation visits reported in Singapore. We found a complex independent relationship between PM variables and AD consultations adjusted for rainfall and absolute humidity. In the subgroup analysis this relationship varied by certain age groups. In general, PM_2.5_ and PM_10_ were positively associated with the number of AD consultation visits while rainfall had a positive association at moderate levels and negative at extremely high levels of precipitation. To our knowledge this is the first study conducted in Singapore investigating the associations between air quality and meteorological factors with the incidence of AD consultation visits.

### Meteorological and air quality effects

We observed a parabolic dose–response relationship for both PM_2.5_ and PM_10_ where AD risk increases up to a threshold and then decreases thereafter. The association between PM_2.5_ and the risk of AD consultation visits was statistically significant up to ~ 110 µg/m^3^, while for PM_10_ the same was observed up to ~ 150 µg/m^3^. As PM_2.5_ is a subset of PM_10_, the associations between PM_10_ and AD consultation visits up to ~ 110 µg/m^3^ can be mainly attributed to PM_2.5_. The higher threshold observed for PM_10_ (up to ~ 150 µg/m^3^) are then likely to be contributed by particles of sizes in the range of 2.5–10 µm in diameter. The mechanism for the parabolic relationship between PM and AD consultation visits likely involves both biological processes as well as human behaviour. The detrimental effects of PM on the exacerbation of AD are well documented. A study found that exposure to PM can lead to the loss of essential structural proteins in the epidermis, including cytokeratin, filaggrin, and E-cadherin^37^. PM was also found to stimulate production of matrix metalloproteinases, inflammatory cytokines by keratinocytes and activate NFκB signalling leading to inflammation in the exposed skin areas^38–40^. In the experimental studies on animals, upon exposure, PM was not only found at the hair follicles but also intracellularly in the barrier-disrupted epidermis. Additionally, PM-exposed skin was found to harbour more *Staphylococcus aureus* colonies that are known to drive the AD disease cycle^41^. Therefore, our findings are consistent with biologically plausible mechanisms of PM-induced AD exacerbation and onset reported in other studies. The threshold effect likely arose from reduced human outdoor activities at extremely high levels of PM (> 100 µg/m^3^ for PM_2.5_) that results in a reduction of the actual exposure to it^42^. Moreover, hourly measures of the Pollutant Standards Index are publicly available and this would have contributed to higher awareness on prevailing PM levels and consequently result in changes in behaviour among Singaporeans. Visual and smell cues from transboundary haze would also have prompted changes in behaviour. In addition, patients with AD are likely to cancel their consultation visits during extreme haze events and stay indoors. Consistent with our findings, positive associations between PM and AD were reported in studies from Korea, Taiwan, and China^15–17^. The detrimental effects of PM on the exacerbation of AD are well documented.

The effect of rainfall on the AD consultation visits was positive for values just above the median and negative for extremely high values. The initial increase in the risk of AD consultation visits above the median rainfall levels may be attributed to the higher concentration of allergens indoors^43^. Similar positive findings were found in a study in Korea^43^. Another study in Nigeria reported higher odds of presenting with AD during dry season compared to wet season, consistent with our finding of lower risk of AD consultation visits at higher levels or rainfall^44^. Additionally, some patients are likely to cancel their appointments during periods of heavy rain, resulting in the decreased risk of AD consultation visits.

Absolute humidity was found to have a negative effect on the number of AD consultation visits for values below and above the median at lag 4 and lag 5 (Fig. S7). There have been conflicting reports on humidity association with AD. While a study in Korea showed humidity to be negatively associated with AD, a study in the USA demonstrated positive associations^16,45^. The authors in the latter study hypothesized that increased sweating in an environment with higher humidity causes irritation due to acidity of the sweat and this may thus promote T helper type 2- and type 17-mediated inflammation^46,47^. The decreased risk of AD consultation visits for high levels of absolute humidity, on the other hand, could be due to behavioural changes in people living in Singapore. The mechanism could be similar to rain and PM where at high humidity people increase the usage of air conditioning indoors, minimizing the actual exposure to humidity^42^.

Although some studies reported associations between AD and other air pollutants like O_3_, and NO_2_ with AD, we did not observe statistically significant effects for these variables in our study^16,17^. This could be due to some synergistic effect of all air pollutants on AD exacerbation or as yet unknown setting-specific differences.

### Gender and age effect modification

Although some studies reported differential effect of air pollutants on AD in males and females, we did not observe statistically significant differences between the sexes^21^. According to Kanda et al.^21^ the differential effect of air pollution arises from hormonal differences between the sexes. However, the study points out that these differences are also specific to different types of ADs. In our study we looked at a cumulative effect of air pollutants on all AD consultation visits, regardless of the types of AD. This could potentially lead to inability to detect differential effects of air pollutants on AD between males and females.

In the age subgroup analysis, we observed statistically significant difference between 18 years old and above and 8–12 years old groups for PM_2.5_. The effect of PM_2.5_ in the 18 years old and above group was similar to the overall effect in the main analysis. For the 8–12 years old groups the effect was largely not statistically significant. One possible reason for the statistically significant difference observed could be due to the greater level of urgency in treatment of AD in children compared to adults. Delays in the AD consultation visits in adults may partially be attributable to work and study commitments whereas greater attention may be given to children by their caregivers. As PM worsens the symptoms of AD when skin barrier is already compromised, delay in managing the disease allows for sufficient time for PM’s detrimental effect to take place. However, this theory is speculative and requires further investigation.

## Strengths and limitations

Although nationally reported AD cases were not available, we included all reports from the largest outpatient dermatological centre in Singapore receiving dermatological referrals from all parts of the country. We have no reason to believe that the effect of air pollutants and meteorological factors would differ for individuals who sought care from other healthcare providers. Therefore, our estimates were likely to reflect the general patterns in the wider community. This is the first study investigating effect of air pollutants and meteorological variables on risk of AD consultation visits conducted in Singapore. The study was conducted on the weekly timescale due to non-availability of the daily outcome data, which would have given higher resolution to the study. The generalization of the mean air pollutant concentrations and weather measures across Singapore may have contributed to the attenuation of our effect estimates. Individual level living conditions, such as the amount of time spent indoors and the use of air purifiers, which may have altered pollutant exposures, were not considered in this study. This study is limited by the granularity of the data, as it cannot distinguish between the effects of weather variables in different geographical locations across Singapore. We were unable to fit all air pollutants in a single model due to collinearity. Nonetheless, we were able to group the air pollutants into different sub-models where appropriate to obtain adjusted risk estimates for independent air pollutant exposures.

The amount of outpatient consultations for AD provides an indirect surrogate measure of the development of eczema symptoms. However, this is also sensitive to other unmeasured factors that can affect health-seeking behaviours. Health-seeking behaviours cannot be easily measured but are believed to relatively stable across the weeks.

## Conclusion

In this study we found statistically significant short-term associations between AD consultation visits with particulate matter and rainfall variability. The associations between PM and AD consultation visits could be explained based on plausible biological mechanisms and human behaviour. Less clear are the associations between meteorological variables and rainfall for which further studies are necessary. Variations in particulate matter and rainfall trends may possibly be used to inform healthcare providers on the anticipated rise in AD cases, with the institution of preventive therapies for patients and adequate allocation of healthcare resources in healthcare institutions.

### Supplementary Information

Supplementary Information.

## Data Availability

The datasets used and/or analysed during the current study available from the corresponding author on reasonable request.
