# Supplementary material for: Association between exposure to ambient air pollution, meteorological factors and atopic dermatitis consultations in Singapore—a stratified nationwide time-series analysis
Source: Sci Rep. 2024 May 6;14:10320. doi: 10.1038/s41598-024-60712-4 (PMC11074125; doi:10.1038/s41598-024-60712-4)
Supplement: Supplementary file 1 — Supplementary Information. [file 41598_2024_60712_MOESM1_ESM.docx]

**Supplementary Material**

Table S1: Pearson correlation between air pollutants.

|  | **PM2.5** | **PM10** | **SO_2_** | **CO** | **NO_2_** | **O_3_** | **AH** | **RF** |
| --- | --- | --- | --- | --- | --- | --- | --- | --- |
| PM2.5 | 1 |  |  |  |  |  |  |  |
| PM10 | **0.98** | 1 |  |  |  |  |  |  |
| SO_2_ | 0.23 | 0.15 | 1 |  |  |  |  |  |
| CO | **0.78** | **0.77** | 0.17 | 1 |  |  |  |  |
| NO_2_ | 0.27 | 0.22 | **0.69** | 0.46 | 1 |  |  |  |
| O_3_ | 0.32 | 0.39 | -0.25 | 0.13 | -0.19 | 1 |  |  |
| AH | - | - | - | - | - | - | 1 | 0.11 |
| RF | - | - | - | - | - | - | 0.11 | 1 |

All correlation coefficients were statistically significant (P < 0.05). AH – Absolute humidity, RF - Rainfall

Bolded values indicate that correlation coefficient exceeds the 0.5 threshold.

Table S2. Model selection and outcomes of LRT test

| **Model** | **Variable** | **LRT test p-value** |
| --- | --- | --- |
|  |  |  |
| **PM_2.5_ model** |  |  |
| Core model | Ns(time, 7 df) | - |
|  | Number of public holidays in a week | - |
|  | Offset(log(population) | - |
| Meteorological variables | Rain (Crossbasis term) | >0.001 |
|  | Absolute humidity (Crossbasis term) | 0.002 |
| Air quality variables | PM_2.5_ (Crossbasis term) | 0.005 |
|  |  |  |
| **PM_10_ model** |  |  |
| Core model | Ns(time, 7 df) | - |
|  | Number of public holidays in a week | - |
|  | Offset(log(population) | - |
| Meteorological variables | Rain (Crossbasis term) | >0.001 |
|  | Absolute humidity (Crossbasis term) | >0.001 |
| Air quality variables | PM_10_ (Crossbasis term) | 0.041 |


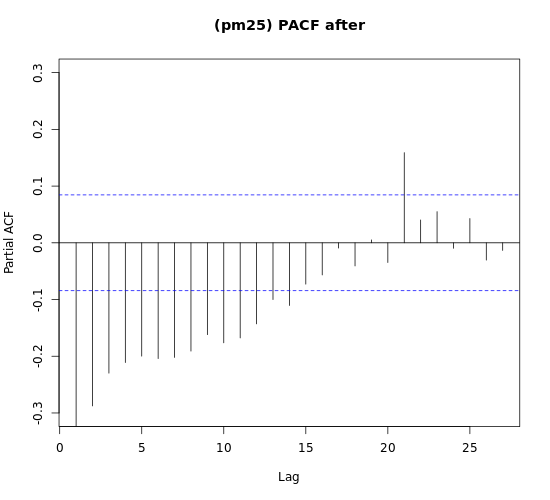

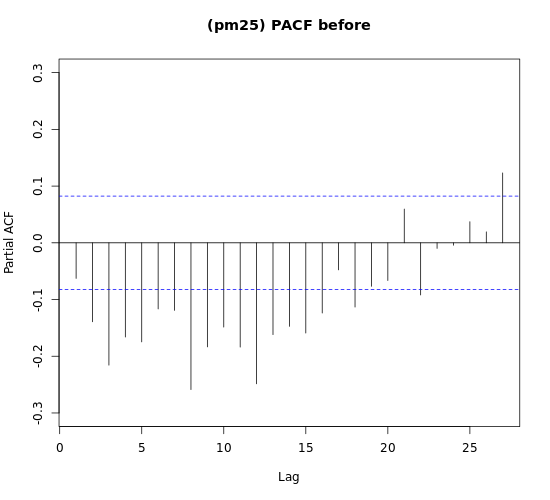


(A)

(B)

Figure S1. Partial autocorrelation function plots for PM_2.5_ model before (A) and after (B) adjustment.


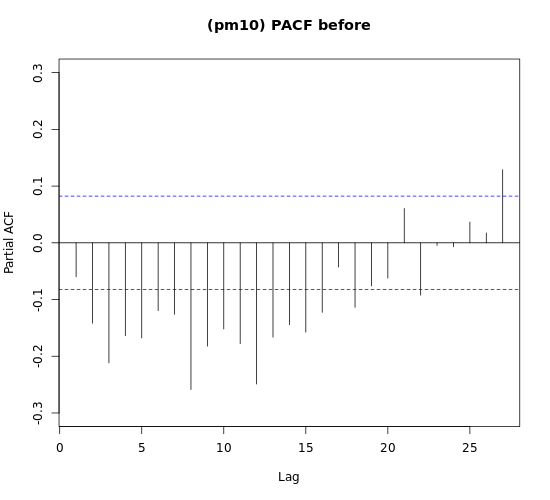

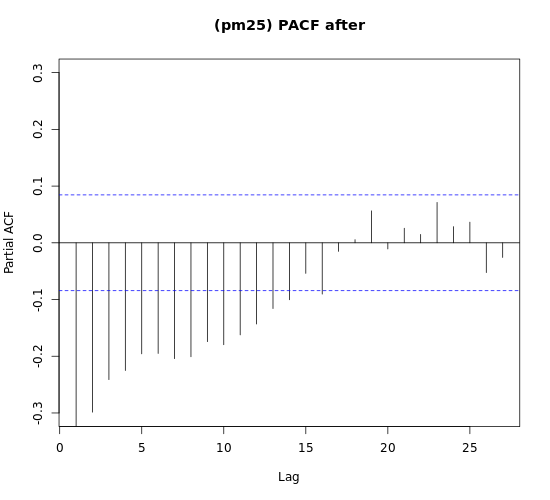


(A)

(B)

Figure S2. Partial autocorrelation function plots for PM_10_ model before (A) and after (B) adjustment.


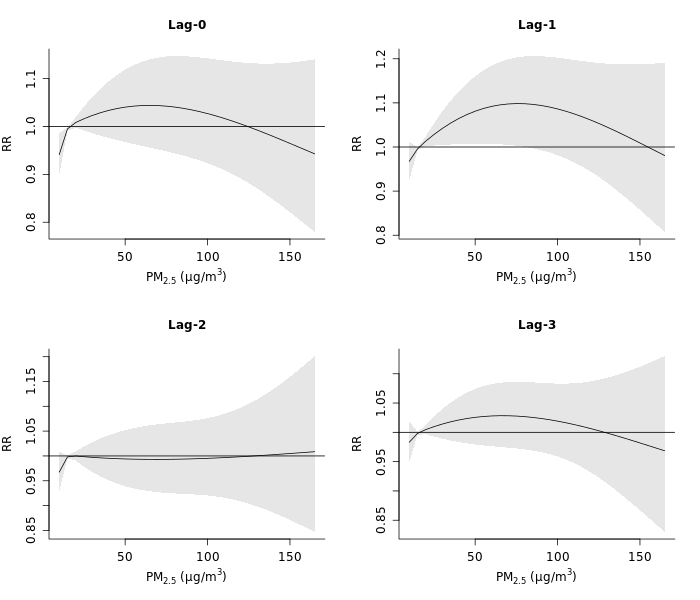

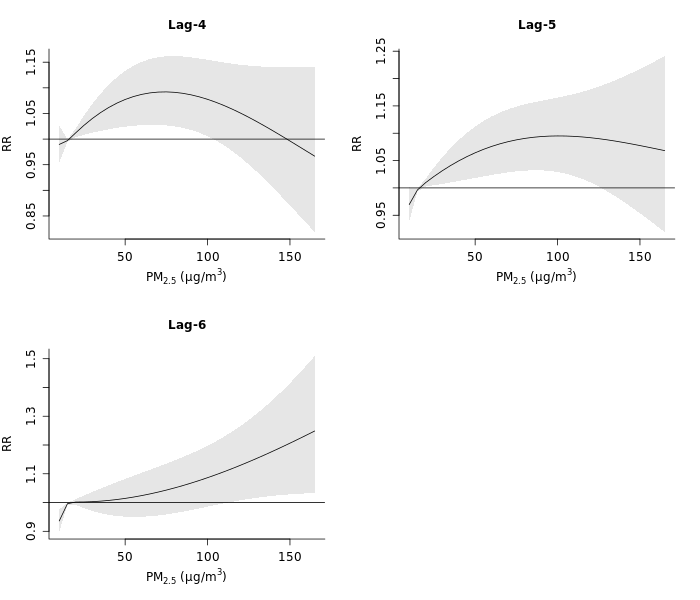


Figure S3. Exposure-response curve showing the effect at specified lags from week 0 to week 6 for PM_2.5_ from PM_2.5_ model. Solid lines represent relative risk (RR), grey shaded areas represent 95% confidence intervals (CIs).


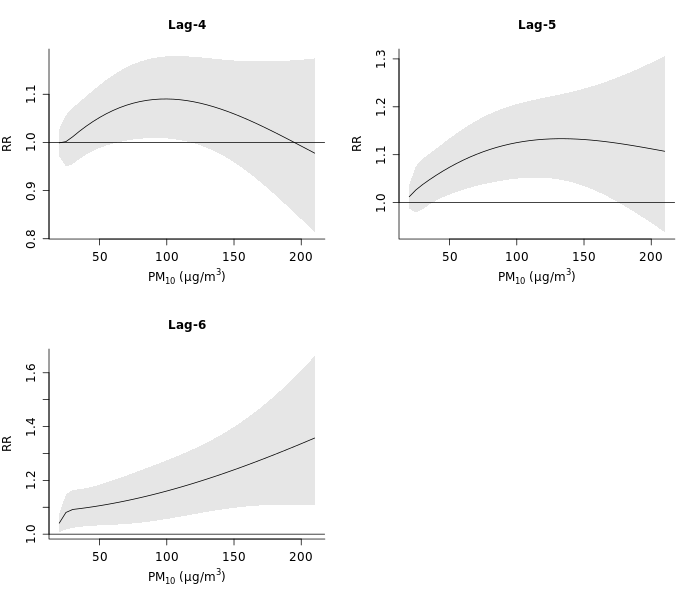

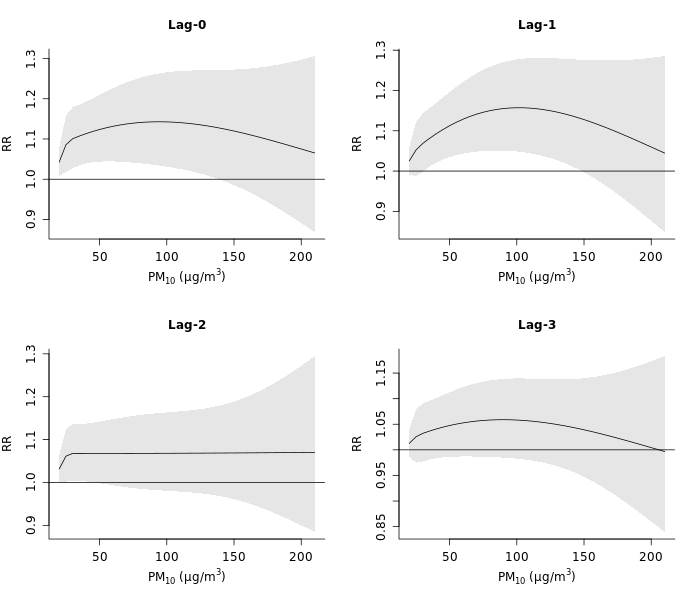


Figure S4. Exposure-response curve showing the effect at specified lags from week 0 to week 6 for PM_10_ from PM_10_ model. Solid lines represent relative risk (RR), grey shaded areas represent 95% confidence intervals (CIs).


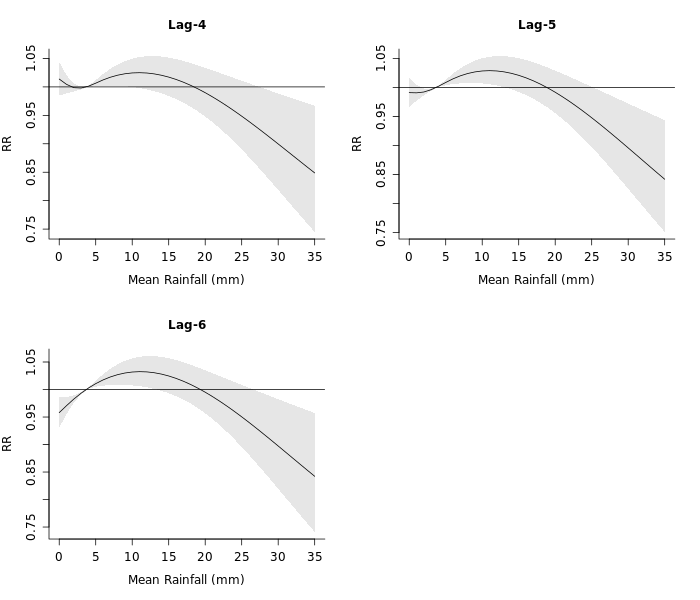

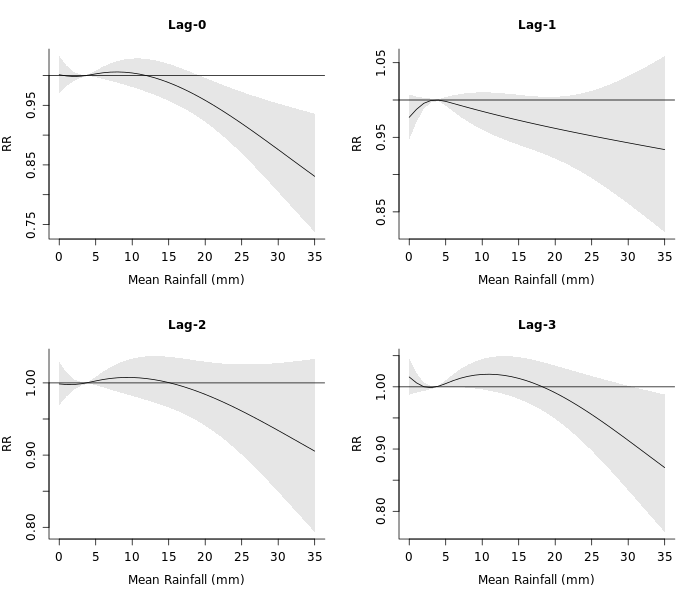


Figure S5. Exposure-response curve showing the effect at specified lags from week 0 to week 6 for mean rainfall from PM_2.5_ model. Solid lines represent relative risk (RR), grey shaded areas represent 95% confidence intervals (CIs).


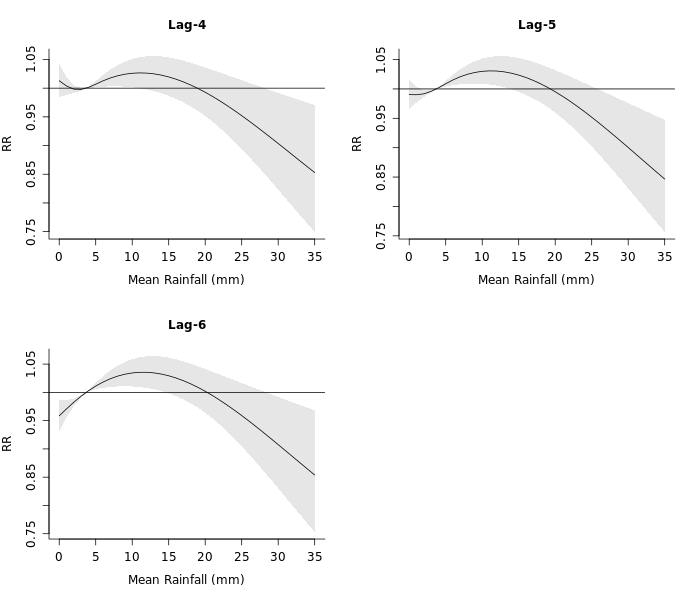

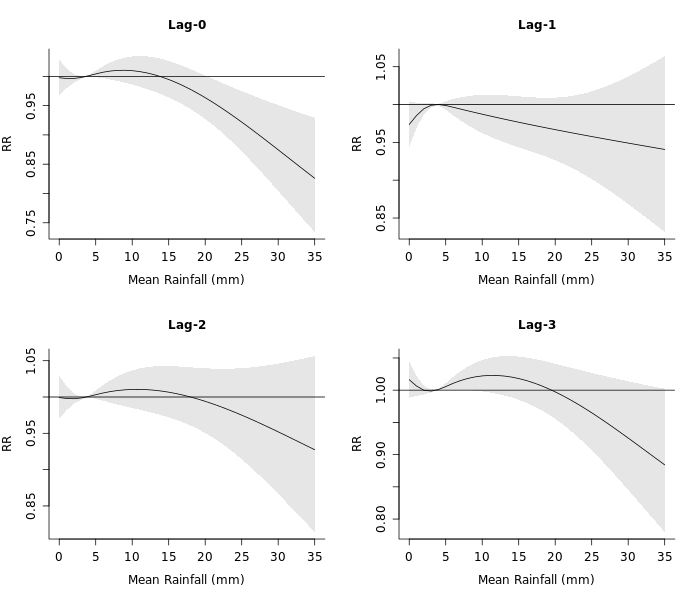


Figure S6. Exposure-response curve showing the effect at specified lags from week 0 to week 6 for mean rainfall from PM_10_ model. Solid lines represent relative risk (RR), grey shaded areas represent 95% confidence intervals (CIs).


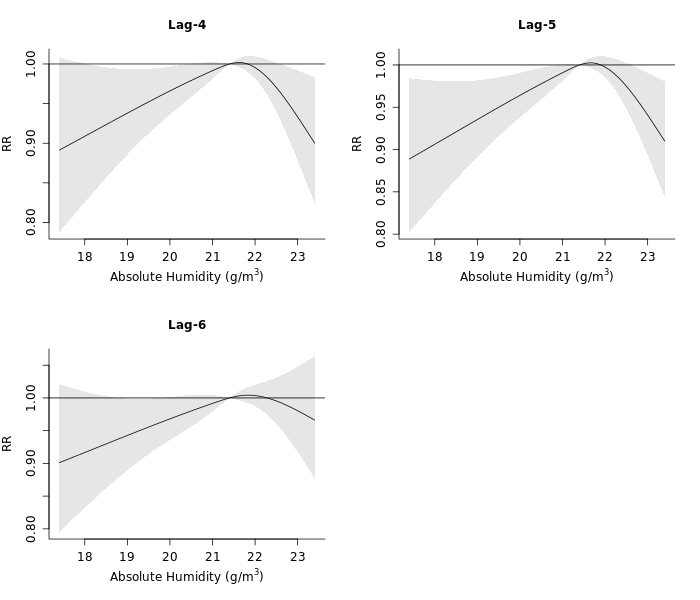

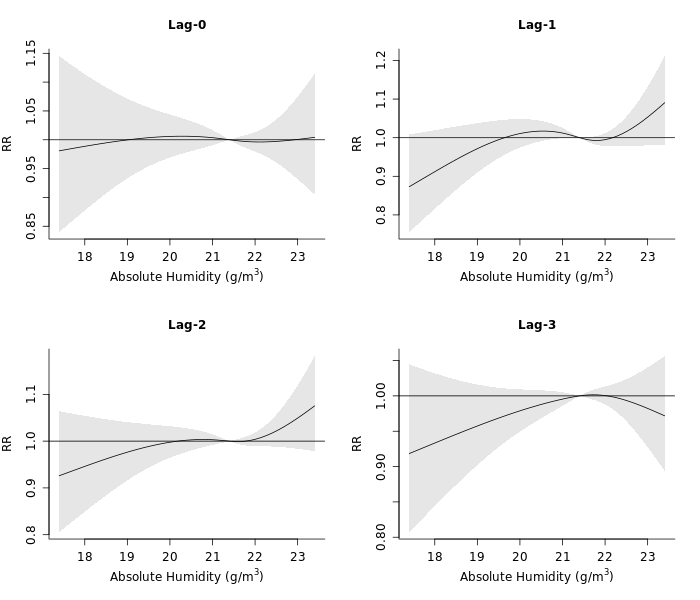


Figure S7. Exposure-response curve showing the effect at specified lags from week 0 to week 6 for absolute humidity from PM_2.5_ model. Solid lines represent relative risk (RR), grey shaded areas represent 95% confidence intervals (CIs).


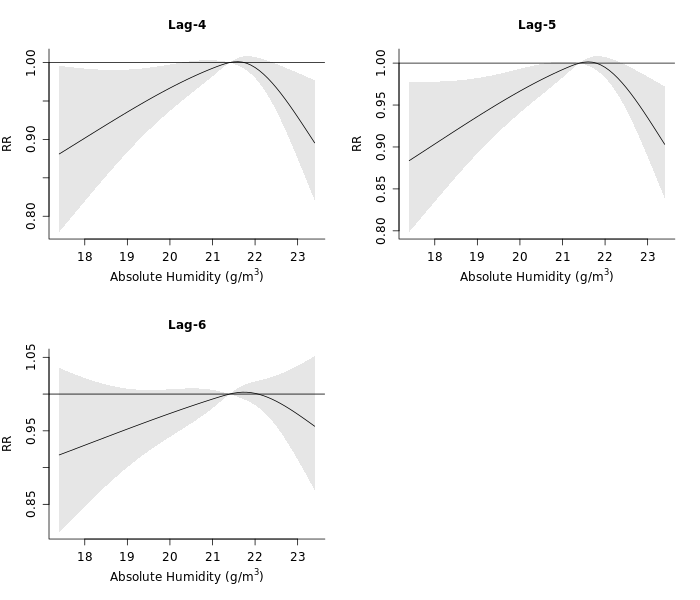

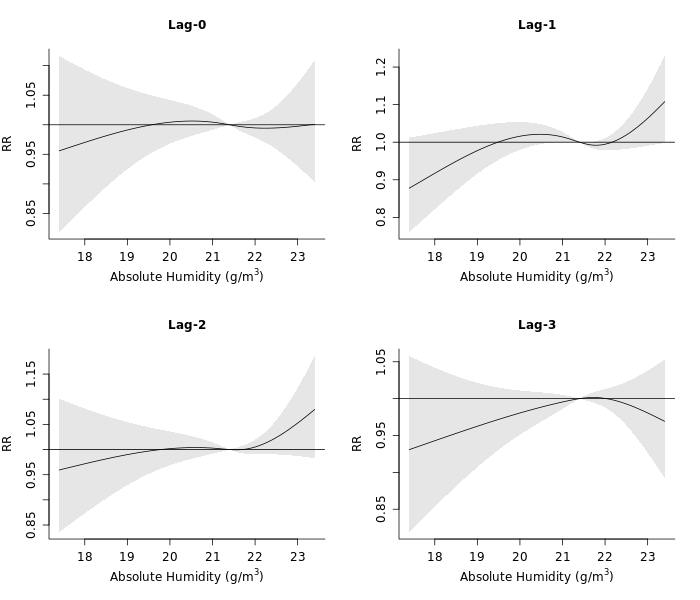


Figure S8. Exposure-response curve showing the effect at specified lags from week 0 to week 6 for absolute humidity from PM_10_ model. Solid lines represent relative risk (RR), grey shaded areas represent 95% confidence intervals (CIs).
